# Validating a Computational Framework for Ionic Electrodiffusion with Cortical Spreading Depression as a Case Study

**DOI:** 10.1523/ENEURO.0408-21.2022

**Published:** 2022-04-22

**Authors:** Ada J. Ellingsrud, Didrik B. Dukefoss, Rune Enger, Geir Halnes, Klas Pettersen, Marie E. Rognes

**Affiliations:** 1 Department for Numerical Analysis and Scientific Computing, Simula Research Laboratory, Oslo 0164, Norway; 2 Letten Centre, Division of Anatomy, Department of Molecular Medicine, Institute of Basic Medical Sciences, University of Oslo, Oslo 0317, Norway; 3 CINPLA, Faculty of Mathematics and Natural Sciences, University of Oslo, Oslo 0316, Norway; 4 Institute of Physics, Faculty of Science and Technology, Norwegian University of Life Sciences, Ås 1432, Norway; 5 NORA, Faculty of Mathematics and Natural Sciences, University of Oslo, Oslo 0316, Norway; 6 Department of Mathematics, Faculty of Mathematics and Natural Sciences, University of Bergen, Bergen 5020, Norway

**Keywords:** computational modelling, cortical spreading depression, ionic electrodiffusion and osmosis, validation

## Abstract

Cortical spreading depression (CSD) is a wave of pronounced depolarization of brain tissue accompanied by substantial shifts in ionic concentrations and cellular swelling. Here, we validate a computational framework for modeling electrical potentials, ionic movement, and cellular swelling in brain tissue during CSD. We consider different model variations representing wild-type (WT) or knock-out/knock-down mice and systematically compare the numerical results with reports from a selection of experimental studies. We find that the data for several CSD hallmarks obtained computationally, including wave propagation speed, direct current shift duration, peak in extracellular K^+^ concentration as well as a pronounced shrinkage of extracellular space (ECS) are well in line with what has previously been observed experimentally. Further, we assess how key model parameters including cellular diffusivity, structural ratios, membrane water and/or K^+^ permeabilities affect the set of CSD characteristics.

## Significance Statement

Movement of ions and molecules in and between cellular compartments is fundamental for brain function. Cortical spreading depression (CSD) is associated with dramatic failure of brain ion homeostasis. Better understanding the sequence of events in CSD could thus provide new insight into physiological processes in the brain. Despite extensive experimental research over the last decades, even basic questions related to mechanisms underlying CSD remain unanswered. Computational modeling can play an important role going forward, since simulation studies can address hypotheses that are difficult to target experimentally. Here, we assess the physiological validity of a novel mathematical framework for detailed modeling of brain electrodiffusion and osmosis, and provide a platform for *in silico* studies of CSD and other cerebral electromechanical phenomena.

## Introduction

Cortical spreading depression (CSD) is a slowly propagating wave of depolarization of brain cells followed by temporary silencing of electrical brain activity because of a complete collapse of cellular ion homeostasis (Pietrobon and Moskowitz, 2014). CSD is characterized by elevated levels of extracellular K^+^ and glutamate (Somjen, 2001), swelling of neuronal somata and dendrites (Takano et al., 2007), swelling of astrocyte endfeet (Rosic et al., 2019), and pronounced shrinkage of the extracellular space (ECS; Mazel et al., 2002). Analyzing the sequence of events in CSD may provide new insight into physiological processes underlying both normal brain function and pathophysiological processes pertinent to a range of brain disorders (Enger et al., 2017).

Despite extensive research over the last decades, even basic questions relating to the mechanisms underlying CSD remain unanswered (Miura et al., 2007). Computational, or *in silico*, modeling may play an important role going forward, not least since simulation studies can address hypotheses and points of debate that are difficult to isolate or address experimentally. Here, we consider a comprehensive computational framework (the Mori framework), describing spatial and temporal dynamics of intracellular and extracellular ion concentrations, electric potentials, and volume fractions (Mori, 2015). The framework has previously been applied to study the roles of glial cells, NMDA receptors and glutamate propagation in CSD (O’Connell and Mori, 2016; Tuttle et al., 2019). However, as simulations have not to any significant extent been compared with experimental findings, the physiological validity of the computational framework remains an open question.

To systematically address this issue, we here simulate CSD in different model scenarios, and compare the computational predictions with values from the experimental literature. Our scenarios mimic different mouse models with varying structural and functional parameters, including varying intracellular diffusion, varying transmembrane water and K^+^ permeabilities, and varying membrane characteristics. These choices of model scenarios are in part motivated by the incomplete or disparate findings on the role of AQP4 (Thrane et al., 2013; Yao et al., 2015; Enger et al., 2017; Rosic et al., 2019), and K_ir_ 4.1 channels (Djukic et al., 2007; Enger et al., 2015; Ohno, 2018), both expressed in the glial cell membranes, in CSD.

Overall, we find that the range of wave speeds, direct current (DC) shift durations, peak in extracellular K^+^, neuronal changes in volume fraction, and ECS shrinkage obtained computationally all overlap with the experimentally reported ranges in wild-type (WT) mice. Further, the intracellular glial diffusivity strongly influences the DC shift, while the ratio of neuronal and glial membrane area-to-tissue volume strongly affects the CSD wave speed. Reducing the glial water permeability has a pronounced effect on cellular swelling, whereas the CSD depolarization wavefront speed and the other quantities of interest remain unaltered. In addition, we find that reducing the K_ir_ 4.1 expression results in reduced glial swelling and depolarization of the glial membrane during CSD.

## Materials and Methods

### Mathematical and computational framework

**Figure 1.**
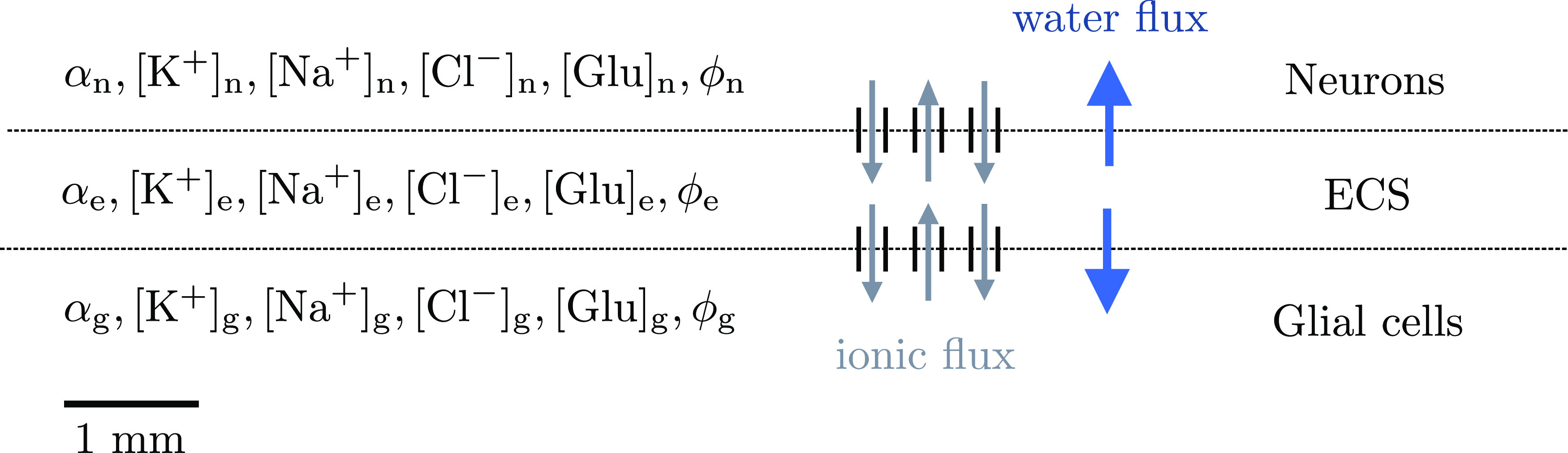
Model overview. The tissue is represented as a 1D domain of length 10 mm including neurons, ECS, and glial cells. Within each compartment, the model describes the dynamics of the volume fraction (*α*), the Na^+^, K^+^, Cl^–^ and glutamate concentrations ( $\left[{\text{Na}}^{+}],\;[{\text{K}}^{+}],\;[{\text{Cl}}^{-}],\;[\text{Glu}\right]$), and the potential (*ϕ*). Communication between the compartments occur via ionic and/or water membrane fluxes.

We model ionic electrodiffusion and osmotic water flow in brain tissue via the Mori framework, as introduced by Mori (2015), studied numerically by Ellingsrud et al. (2021), and applied by O’Connell and Mori (2016) and Tuttle et al. (2019). This framework describes tissue dynamics in an arbitrary number of cellular compartments and the ECS via coupled ordinary differential equations and partial differential equations in a model domain. Here, we consider a version of the general framework presented by Mori (2015): we use the set of equations and physical parameters introduced by Tuttle et al. (2019). Specifically, we consider three compartments representing neurons, glial cells and the ECS (see Fig. 1), and induce CSD computationally in a one-dimensional model domain of length 10 mm. In particular, we consider the glial subtype astrocyte (for convenience, we will henceforth use the terms glia and astrocyte interchangeably). The model predicts the evolution in time and distribution in space of the volume fraction *α*, the electrical potential *ϕ*, and the concentrations $\left[{\text{Na}}^{+}],[{\text{K}}^{+}],[{\text{Cl}}^{-}],[\text{Glu}\right]$ in each of these compartments. The membrane potential is defined as the difference between the intracellular and extracellular potentials, both for the neurons and the glial cells.

Interaction between the compartments is modelled by exchange across the neuron-extracellular and glia-extracellular membranes. In terms of osmotic water flow, the water permeability and area-to-volume ratio of the cellular membranes are key model parameters. To account for transmembrane ion movement across the neuron-extracellular membrane, we consider leak channels, voltage-gated K^+^ and Na^+^ channels, NMDA receptors, and the Na^+^/K^+^-ATPase. Across the glia-extracellular interface, we model leak channels, the inward rectifying K^+^ channel K_ir_ 4.1, the Na/K/Cl co-transporter, and the Na^+^/K^+^-ATPase. The precise mathematical formulation is detailed in Tuttle et al. (2019), and for the sake of completeness included in Extended Data 1.

10.1523/ENEURO.0408-21.2022.ed1 Extended Data 1 Supplementary material. Download Extended Data 1, TEX file.

### Triggers of CSD and spreading depolarization (SD)

CSD can be triggered in various ways experimentally: by electrical stimulation on the surface of the cerebral cortex (Leao, 1944), pinprick (Richter et al., 2002; Rosic et al., 2019), or by topical application of K^+^ (Yao et al., 2015; Enger et al., 2017). The consequent wave of SD is followed by a period of depressed spontaneous neuronal activity. Notably, such spontaneous activity may already be extinguished in metabolically compromised tissue (Dreier, 2011; Lauritzen et al., 2011). Therefore, the term SD is used to denote the phenomenon in the setting of hypoxia and ischemia (Somjen, 2001; Dreier, 2011). As opposed to in CSD, the SD associated conditions, such as traumatic brain injury, ischemic stroke and intracranial hemorrhage, are associated with ATP depletion and a consequent failure of the Na^+^/K^+^-ATPase (Lauritzen et al., 2011; Cozzolino et al., 2018). One notable difference between CSD and SD is the time course of recovery: the restoration of ionic gradients, repolarization of the tissue and recovery of brain function typically take longer in SD than in CSD, depending on the degree of local metabolic compromise and impairment of Na^+^/K^+^-ATPase activity (Pietrobon and Moskowitz, 2014). All these scenarios result in a local extracellular K^+^ increase, which in turn causes opening of voltage gated cation channels. Here, we consider three different mechanisms for triggering CSD and SD. Specifically, the first two mechanisms trigger CSD, the latter SD:

- Excitatory fluxes: a flux of Na^+^, K^+^, and Cl^–^ is introduced over the neuronal membrane at the first (left-most) 0.02 mm of the computational domain for the first 2 s of simulation time.
- Topical application of K^+^: the initial values for the extracellular K^+^ and Cl^–^ concentrations are increased in the first 1 mm of the computational domain.
- Disabled Na^+^/K^+^-ATPase: Na^+^/K^+^-ATPase is disabled by setting the neuron and glia maximum pump rates to zero in the first 1 mm of the computational domain for the first 2 s of simulation time.

The precise expressions are detailed in Extended Data 1.

### Quantities of interest

Experimental studies of CSD have reported on the speed of CSD waves, the duration and amplitude of extracellular K^+^ and glutamate rises, the DC shift, neuronal and glial swelling, as well as extracellular shrinkage. To compare computational results to the experimental findings, we define the following quantities of interest.

- Mean wave propagation speed (mm/min): given the point *x_i_* at which the neuron potential peaks at time *t_i_*, we define the wave speed *v_i_* as $v_{i}=(x_{i}-x_{i-1})/(t_{i}-t_{i-1})$ at all times *t_i_* for which the neuron potential $\phi_{\text{n}}(x_{i})$ has passed a depolarization threshold of –20 mV and after the wave is fully initiated. We then set the mean wave propagation speed $\bar{v}$ as the average of the *v_i_*.
- Duration and amplitude of the DC shift: we define the DC shift in terms of the change in extracellular potential as follows. The amplitude of the DC shift is the maximal spatial difference in the extracellular potential (sampled at *t* = 60 s). The duration of the DC shift is the difference between the latest and earliest time for which the extracellular potential is below a threshold of –0.05 mV from baseline (sampled at *x* = 1 mm).
- Duration and amplitude of the neuronal, glial and extracellular swelling: these are defined analogously as the duration and amplitude of the DC shift. The lower threshold for neuronal and glial swelling and extracellular shrinkage is set at 0.5%. Shrinkage is defined as negative swelling.
- Duration and amplitude of elevated extracellular K^+^, Cl^–^, and glutamate: these are defined similarly as for the DC shift, with lower thresholds of 8 mm for K^+^ and 002 mm for glutamate, and an upper threshold of 111 mm for Cl^–^.
- Duration and amplitude of neuronal and glial membrane depolarization: these are defined similarly as the above with a lower threshold of –66 mV for the neuronal membrane potential and –77 mV for the glial membrane potential.

In addition to these specific quantities of interest, we plot snapshots in time (i.e., plots of the respective fields vs the spatial coordinate *x*) and the time evolution of the computed fields evaluated at *x *=* *1.0 mm.

### Computational model and model variations

As a baseline, we define a WT model (Model A; see Table 1) with default model parameters (listed in Extended Data 1). In addition, we consider four classes of model variations as described in the following (see also Table 1). Several parameters in the WT model (Model A) are associated with substantial uncertainty, in particular the strength of the glial gap junction coupling and the ratio of membrane area to tissue volume. As such, we present variations of the WT model (Model B and Model C), where we vary these two parameters gradually to explore the response in the CSD wave characteristics. Further, we introduce model versions where vary the glial transmembrane water and K^+^ permeabilities, motivated by the incomplete or disparate findings on the role of AQP4 (Thrane et al., 2013; Yao et al., 2015; Enger et al., 2017; Rosic et al., 2019), and K_ir_ 4.1 channels (Djukic et al., 2007; Enger et al., 2015; Ohno, 2018).

**Table 1:** Overview of the computational models with parameter values: χg: glial gap junction factor, γne: neuronal membrane area-to-volume, γge: glial membrane area-to-volume, ηge: glial membrane water permeability, $g^{{\text{K}}_{\text{ir}}4.1}$**: glial Kir 4.1 resting conductance**

Model	*χ*_g_	*γ*_ne_ (m^– 1^)	*γ*_ge_ (m^– 1^)	*η*_ge_ (m^4^/(mol s))	$g^{{\text{K}}_{\text{ir}}4.1}$ (S/m^2^)
A	0.05	5.38 × 10^5^	6.38 × 10^5^	5.40 × 10^– 10^	1.30
B	0	–	–	–	–
C	–	1.35 × 10^5^	1.6 × 10^5^	–	–
D	–	–	–	0	–
E	–	–	–	–	0.91

Model A corresponds to the default parameters (given in Extended Data 1, only three significant digits included here). The dash (–) indicates no change from the default values.

#### Reduced glial gap junctions

Interconnected astrocytes form syncytia (networks) by gap junctions. Intercellular transport through the astrocytic networks likely facilitate the removal of excess extracellular K^+^ through spatial buffering in the hippocampus (Wallraff et al., 2006). In our computational models, the glial gap junction factor *χ_g_* represents glial gap junctions and defines the effective intercellular diffusion through the astrocytic networks. To explore how diffusion through astrocytic networks affects the CSD wave, we consider a version of the WT model with no glial gap junctions (*χ*_g_ = 0, i.e., a 100% reduction) and as a consequence, a zero effective diffusion coefficient in the glial compartment (Model B). In addition, we examine the graded response of intercellular glial diffusion by reducing the glial gap junction factor *χ*_g_ by 25%, 50%, and 75%.

#### Reduced membrane area-to-volume

The ratio of membrane area to tissue volume (membrane area-to-volume) for the neuronal and glial compartments are averaged model parameters ( $\gamma_{\text{ne}},\;\gamma_{\text{ge}}$) that may be difficult to determine experimentally and that are associated with substantial uncertainty. Kager et al. (2000) report an estimated neuronal membrane area-to-volume based on measurements of a rat hippocampal CA1 neuron, and O’Connell and Mori (2016) assume the same value for the glial membrane area-to-volume. Halnes et al. (2019) independently estimate a higher neuronal and glial area-to-volume value. To explore the effect of reducing the neuronal and glial membrane area-to-volume, we consider model versions where we gradually reduce the area-to-volume parameters by 25%, 50%, and 75% (Model C).

#### Reduced glial membrane water permeability

To study the role and effect of water movement across the astrocytic membrane on CSD dynamics, we define a model variant (Model D) by setting the water permeability of the glial membrane *η*_ge_ to 0 (a 100% reduction). This model thus stipulates that water cannot cross the glial membrane; neither via the lipid bilayer itself nor other membrane mechanisms such as AQP4 channels, VRAC, NKCC, or other co-transporters. As such, it can be viewed as an extreme case providing an upper bound on the effect of, e.g., reduced AQP4 expression on CSD wave characteristics, and allows for comparing computational predictions with the relatively large body of literature on AQP4 in CSD (Thrane et al., 2013; Yao et al., 2015; Enger et al., 2017; Rosic et al., 2019). Note that the water permeability is reduced while keeping all other model parameters constant, and as such, Model D does account for any potential physiological compensatory mechanisms. In addition to the extreme case where the glial water permeability *η*_ge_ is set to zero, we let *η*_ge_ be reduced by 25%, 50%, and 75%.

#### Reduced K_ir_ 4.1 expression

To study the effect of potassium movement across the astrocytic membrane on the CSD dynamics, we define a model variant (Model E) by reducing the K_ir_ 4.1 resting conductance of the glial membrane $g^{{\text{K}}_{\text{ir}}4.1}$. Specifically, we reduce $g^{{\text{K}}_{\text{ir}}4.1}$ by 10%, 20%, 30% (Model E), 40%, 50%, 60%, 70%, and 80%. For K_ir_ 4.1 resting conductance reductions by >30%, the computational (nonlinear) solver fails. Thus, Model E represent a partial K_ir_ 4.1 knock-down. Changes in the membrane parameters affect the steady state of the models, and in response, we also consider new initial states of the system for these model variations (see Extended Data 1).

### Numerical solution and verification

We apply the solution algorithm presented previously (Ellingsrud et al., 2021) and approximate the mathematical model numerically by a finite element method in space, a BDF2 scheme in time and a Strang splitting method. We use spatial and temporal discretization sizes of Δ*x *=* *1.25 μm and Δ*x *=* *3.125 ms, respectively. Numerical verification tests have been conducted to ensure convergence of solutions. The numerical error in the calculated mean wave propagation speed is estimated to be <1.5%, whereas for the other quantities of interest we expect the numerical errors to be negligible (Ellingsrud et al., 2021).

### Validation principles

We perform a quantitative and qualitative comparison of experimental and computational results via (a selection of the) quantities of interest listed above (Quantities of interest). We collected experimental findings from a set of recent experimental studies on CSD (Lauritzen and Hansen, 1992; Theis et al., 2003; Padmawar et al., 2005; Takano et al., 2007; Chang et al., 2010; Zhou et al., 2010; Thrane et al., 2013; Enger et al., 2015, 2017; Yao et al., 2015; Kucharz et al., 2017). For the comparison, we define intervals of computational and experimental values for each of the quantities of interest reported in (a subset of) the experimental studies (wave speed, DC shift and duration, peak extracellular K^+^ concentration, neuronal swelling and ECS shrinkage, elevated extracellular glutamate duration). The experimental ranges are defined by the 25th percentile (Q1, splits off the lowest 25% of the data from the highest 75%) and the 75th percentile (Q3, splits off the highest 25% of the data from the lowest 75%) of the mean values reported in the experimental studies. The computational ranges are defined by the 25th and 75th percentiles of the values from Models A, B, and C. To indicate variability outside the 25th and 75th percentiles, we define minimum and maximum values (the min/max-range) by the lowest and highest data points still within 1.5 IQR (where IQR = Q3 – Q1) of the lower and upper quartiles, respectively. Values outside the min/max-range are taken to be outliers. We qualitatively classify the match between computational and experimental results as follows: in good agreement if the computational and experimental intervals overlap, overlap in range if the min/max-ranges overlap but the intervals do not overlap, and not in agreement if the min/max-ranges do not overlap.

### Calculating changes in volume fractions relative to baseline

Figures and tables shown in Results display the change in volume fraction relative to baseline ( $\Delta \alpha_{r}=\frac{\alpha_{r}-\alpha_{r}^{0}}{\alpha_{r}^{0}}\times 100\%$). We remark that the volume fractions (*α_r_*, r = {*n*, *g*, *e*}) sum to 1 (cf. model equations in Extended Data 1), and thus that total volume is conserved in the computational model.

### Code accessibility

The simulation software described in the paper is freely available online at https://bitbucket.org/adaje/supplementary-code-validating-a-computational-framework-for/src/master/. The code is available as Extended Data 2. The simulations were run in serial on a Lenovo ThinkPad Carbon X1 11th Gen 2.80 GHz × 8 Intel Core i7-1165G7 CPU with Ubuntu 20.04 using FEniCS 2019.1.0, a software library enabling automated solution of (partial) differential equations.

10.1523/ENEURO.0408-21.2022.ed2 Extended Data 2 Code. Download Extended Data 2, ZIP file.

## Results

### Excitatory fluxes trigger wave of depolarization, ionic changes, and swelling

**Figure 2.**
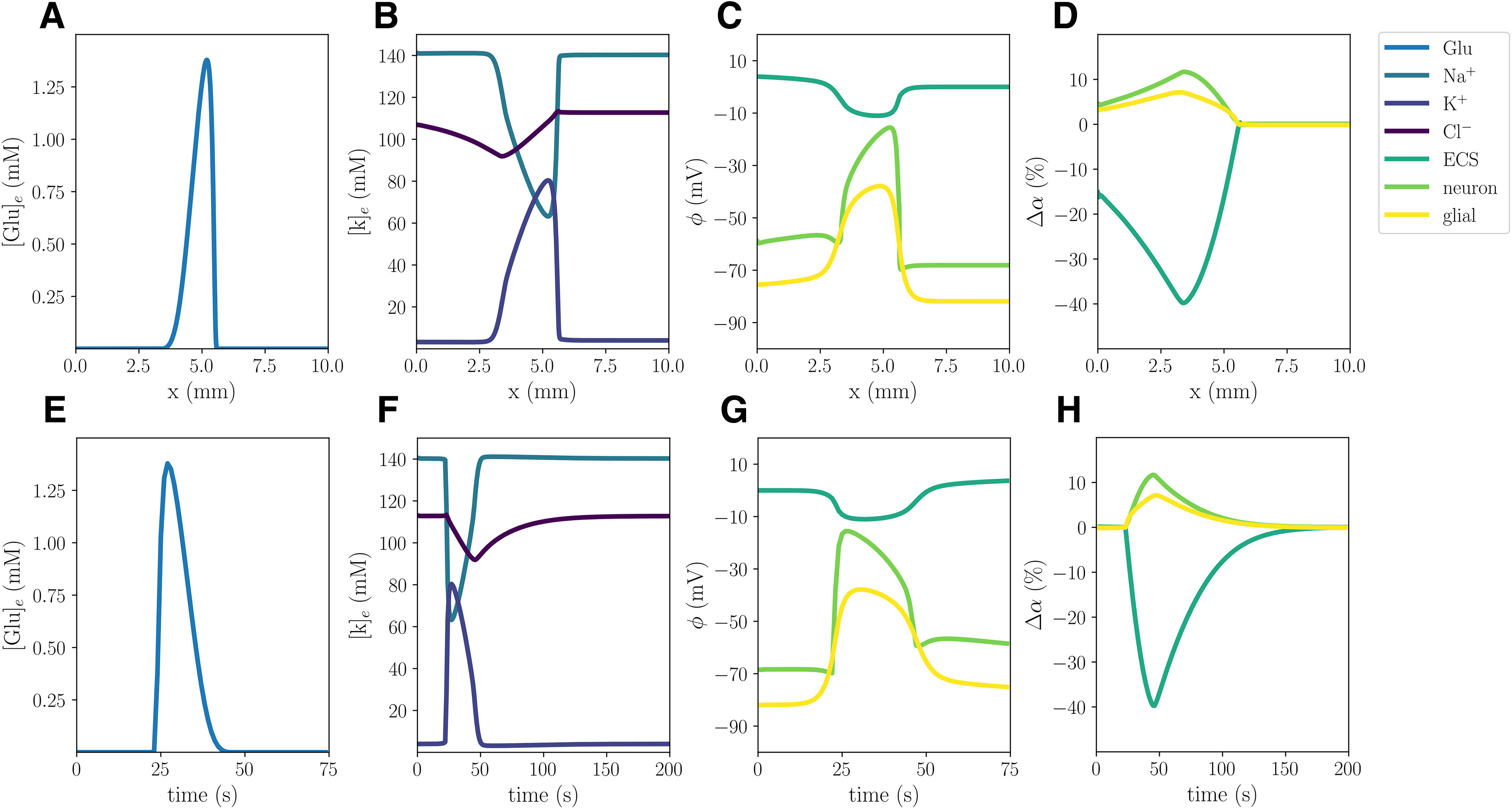
Simulated CSD wave in the WT model (***A***) triggered by excitatory fluxes. The upper panels display a snapshot in time (plot of the respective field vs spatial coordinate *x*) of ECS glutamate (***A***), ECS ion concentrations (***B***), potentials (***C***), and change in volume fractions (***D***) at 60 s. The lower panels display time evolution of ECS glutamate (***E***), ECS ion concentrations (***F***), potentials (***G***), and change in volume fractions (***H***) at *x *=* *2.0 mm.

In the WT computational model (Model A), the excitatory fluxes trigger a wave of neuronal and glial depolarization, changes in ionic concentrations and cellular swelling spreading through the tissue domain (Fig. 2). We observe a depolarization of the neuronal and glial potentials from –68.5 to –15.5 and –82.0 to –38.9 mV, respectively, accompanied by a DC shift with an amplitude of 11.02 mV, with a duration of 32 s (Fig. 2*C*,*G*). The neuronal depolarization wave is followed by an increase in the concentrations of extracellular K^+^ of 76.4 mm (Fig. 2*B*,*F*), and glutamate of 1.38 mm (Fig. 2*A*,*E*), and decreases in extracellular Na^+^ and Cl^–^ concentrations (Fig. 2*B*,*F*). The increased levels of extracellular K^+^ and glutamate persist for 26 and 20 s, respectively, whereas the drop in extracellular Cl^–^ lasts for 84 s. In response to the ionic shifts, both the neurons and the glial cells swell: we observe a neuronal swelling of 11.7% and a glial swelling of 7.13%; the ECS shrinks correspondingly. We observe altered volume fractions for 103–135 s (Table 2). Finally, the wave front has reached 5.25 mm after 60 s, and we observe a mean wave propagation speed of 5.84 mm/min. We note that the wave speeds *v_i_* used to calculate the mean wave propagation speed, varies between 5.78 and 5.85 mm/min.

**Table 2:** Summary of computational quantities of interest for different models (A, B, C, D, E)

Quantity of interest	A	B	C	D	E
Mean wave propagation speed (mm/min)	5.84	5.52	3.19	5.83	5.27
DC shift (mV)	11.02	3.80	10.18	11.02	10.16
DC shift duration (s)	32	50	86	32	36
Neuronal swelling (%)	11.69	11.67	5.69	14.93	11.81
Glial swelling (%)	7.13	7.05	3.74	0	6.73
ECS shrinkage (%)	39.79	39.56	19.32	37.33	39.60
Neuronal swelling duration (s)	109	111	183	115	100
Glial swelling duration (s)	103	101	181	0	148
ECS shrinkage duration (s)	135	135	185	136	141
ECS K^+^ elevation (mm)	76.39	76.46	54.40	75.39	76.60
ECS Glu elevation (mm)	1.38	1.38	0.20	1.37	1.39
ECS Cl^–^ elevation (mm)	21.12	20.88	7.43	23.12	20.81
ECS K^+^ elevation duration (s)	26	26	60	26	27
ECS Glu elevation duration (s)	20	19	25	20	20
ECS Cl^–^ elevation duration (s)	84	84	178	87	90
Neuronal membrane potential (mV)	63.36	63.38	51.51	63.32	64.53
Glial membrane potential (mV)	55.14	55.77	46.52	54.24	48.23
Neuronal membrane potential duration (s)	79	79	158	83	69
Glial membrane potential duration (s)	45	40	96	42	160

Numerical errors are <1.5%.

### Different computational CSD and SD triggers give comparable wave characteristics

CSD and SD can be triggered by different mechanisms experimentally, and we find that the same holds computationally. All three triggering mechanisms considered here, excitatory fluxes, topical application of K^+^ and disabled Na^+^/K^+^-ATPase (see Materials and Methods), induce a propagating SD wave with nearly identical wave characteristics, including a mean wave propagation speed of 5.84, 5.82, and 5.83 mm/min, respectively.

### Reduced glial intercellular diffusion reduces DC shift but maintains membrane potentials

**Figure 3.**
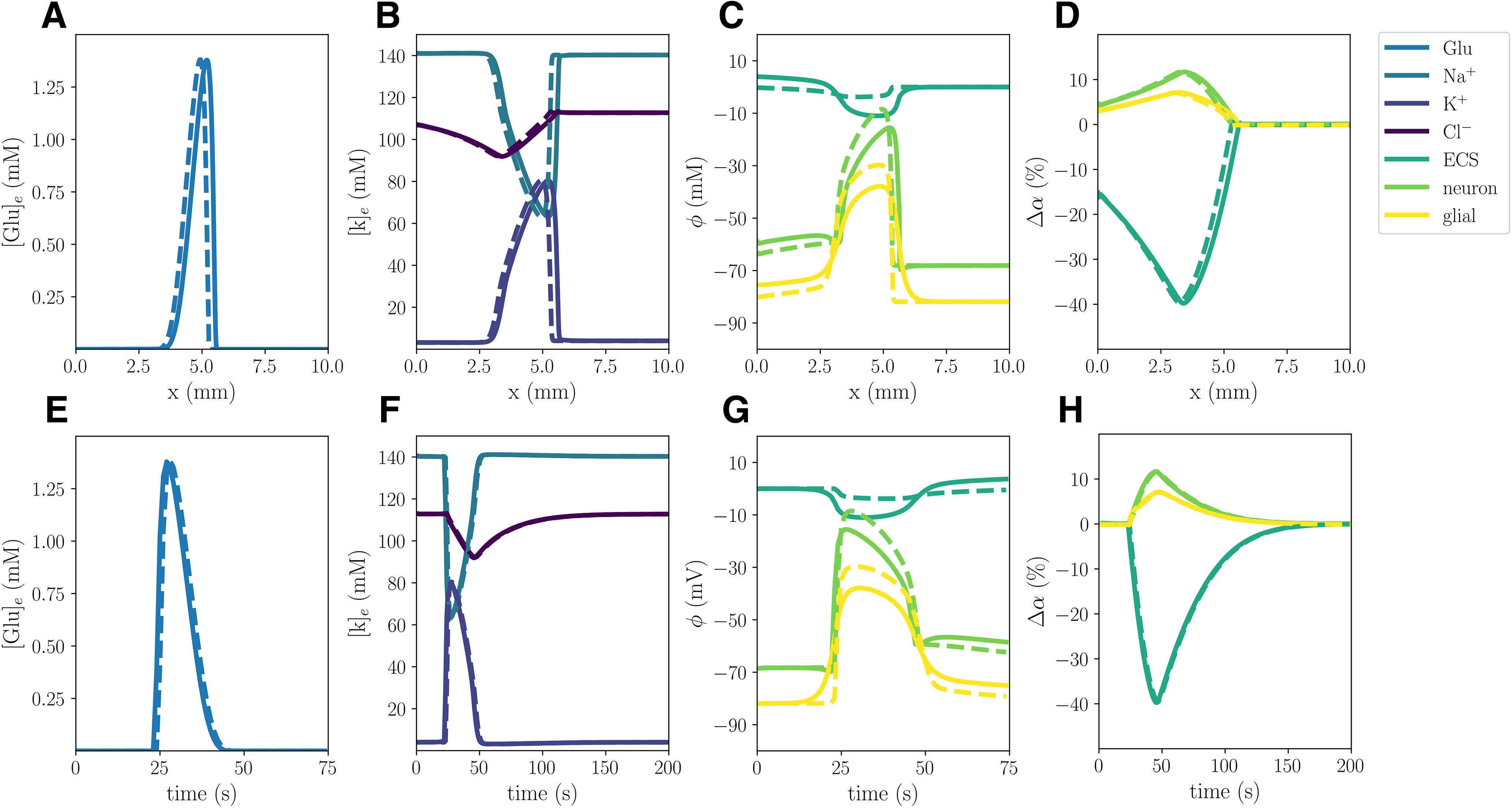
Comparison of Model A (solid) and Model B (stippled) CSD wave. The upper panels display snapshots (plot of field vs spatial coordinate *x*) of ECS glutamate (***A***), ECS ion concentrations (***B***), potentials (***C***), and change in volume fractions (***D***) at 60 s. The lower panels display time evolution of ECS glutamate (***E***), ECS ion concentrations (***F***), potentials (***G***) and change in volume fractions (***H***) evaluated at *x *=* *2.0 mm.

**Figure 4.**
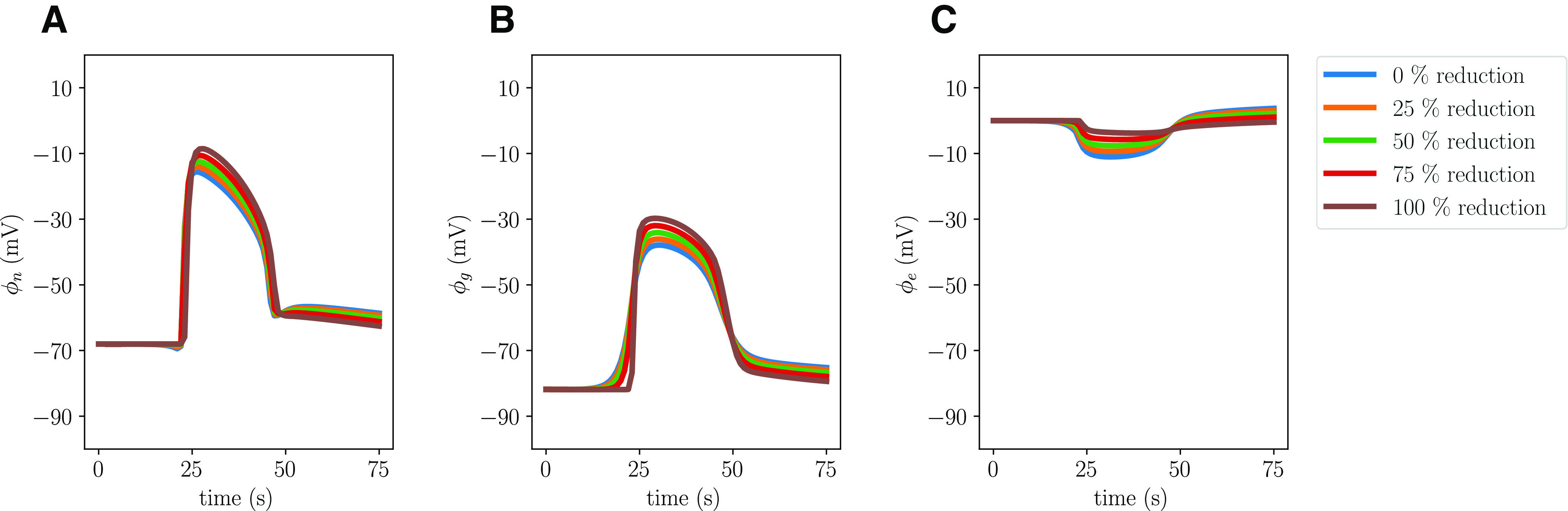
Comparison of electrical potentials during simulated CSD where the glial gap junction factor *χ*_g_ is reduced by 0% (Model A), 25%, 50%, 75%, and 100% (Model B). The panels display neuronal potentials (***A***), glial potentials (***B***), and ECS potentials (***C***) evaluated at *x *=* *2.0 mm.

The glial gap junction factor *χ_g_* modulates the intercellular diffusion through the astrocytic networks. Reducing the effective intercellular diffusion (Model B) does not lead to substantial changes in the CSD wave speed, ionic concentration changes, or cellular swelling (Table 2, Model A vs B; Fig. 3*C*,*G*): Model B gives a 5% reduction in the mean wave propagation speed (to 5.52 mm/min), and <2% in the other (ionic concentration or cellular swelling) quantities of interest. On the other hand, the DC shifts notably differ: the DC shift amplitude of Model B (3.80 mV) is 66% smaller than in Model A (11.02 mV). Yet, we observe that both the glial and neuronal potentials depolarize more in Model B compared with Model A, and thus the membrane potentials do not differ substantially between the two models. In the case of 25%, 50%, and 75% reductions in the effective intercellular diffusion, we observe a similar behavior: the DC shift amplitude gradually decreases as the glial gap junction factor is decreased, whereas the neuronal and glial depolarizations gradually increase (Fig. 4).

### Reduced membrane area-to-volume reduces CSD wave speed and amplitudes

Different values for the ratios of cell membrane area to unit tissue volume *γ* in brain tissue have been reported in the literature (Kager et al., 2000; Halnes et al., 2019). These parameters are thus uncertain and it is key to understand their effect on CSD wave characteristics.

**Figure 5.**
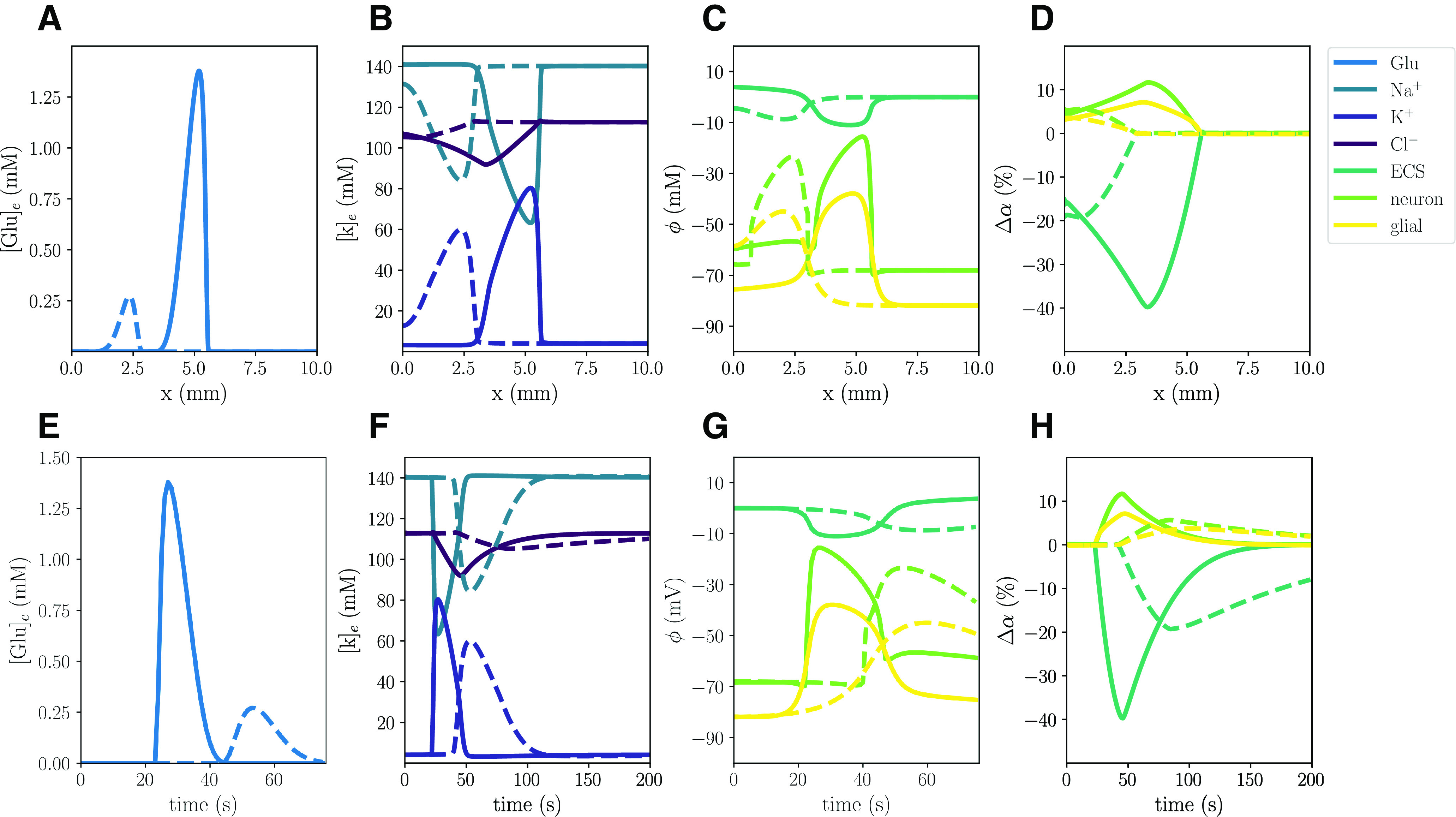
Comparison of Model A (solid) and Model C (stippled) CSD wave. The upper panels display snapshots (plot of field vs spatial coordinate *x*) of ECS glutamate (***A***), ECS ion concentrations (***B***), potentials (***C***), and change in volume fractions (***D***) at 60 s. The lower panels display time evolution of ECS glutamate (***E***), ECS ion concentrations (***F***), potentials (***G***), and change in volume fractions (***H***) evaluated at *x *=* *2.0 mm.

Reducing the membrane area-to-volume for the neurons *γ*_ne_ and glial cells *γ*_ge_ by 75% (Model C) substantially alters the CSD wave characteristics (Fig. 5). In particular, the amplitudes of the ECS K^+^ and glutamate elevations are reduced by 29% and 86%, respectively. We observe that both neurons and glial cells swell less, and correspondingly the ECS shrinks less. Further, the amplitudes of the neuronal and the glial membrane potentials are, respectively, 18% and 16% smaller in the Model C than in the Model A (Table 2). Remarkably, the reduced membrane area-to-volume substantially slows down the CSD wave: the wave speed is 45% slower in Model C (3.19 mm/min) compared with Model A (5.84 mm/min; Table 2).

**Figure 6.**
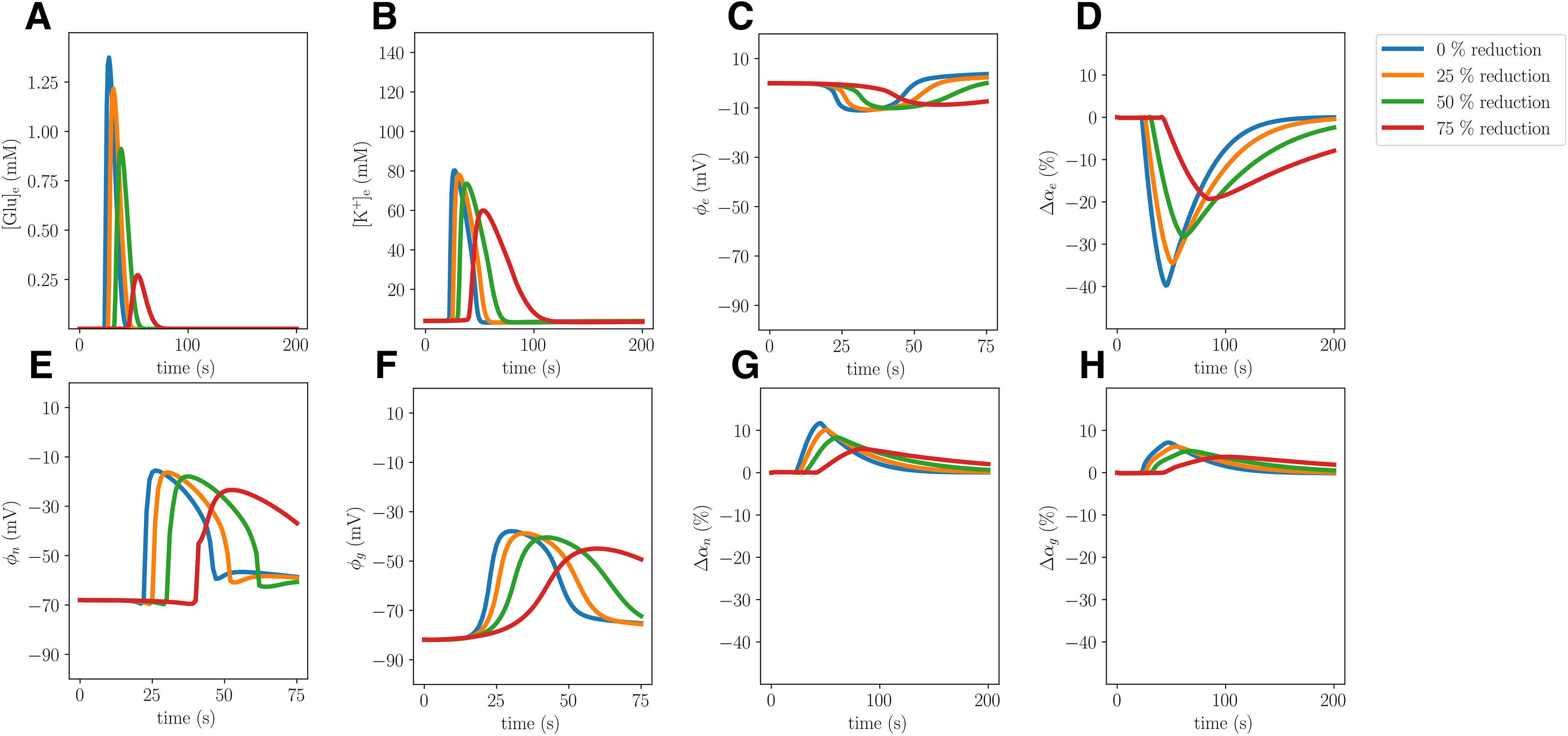
Comparison of simulated CSD where the neuronal and glia membrane area-to-volume factors *γ*_ne_ and *γ*_ge_ are reduced by 0% (Model A), 25%, 50%, and 75% (Model C). The panels display ECS glutamate concentrations (***A***), ECS potassium concentrations (***B***), ECS potentials (***C***), change in ECS volume fractions (***D***), neuronal potentials (***E***), glial potentials (***F***), change in neuronal volume fractions (***G***), and change in glial volume fractions (***H***) evaluated at *x *=* *2.0 mm.

Gradually reducing the membrane area-to-volume for the neurons *γ*_ne_ and the glial cells *γ*_ge_ (by 25%, 50%, and 75%) results in a gradual reduction in the amplitudes of the ECS K^+^ and glutamate elevations, the neuronal and glial swelling, the amplitudes of the neuronal and the glial membrane potentials, and the wave propagation speed (Fig. 6).

### Computational versus experimental quantities of interest in WT mice

**Figure 7.**
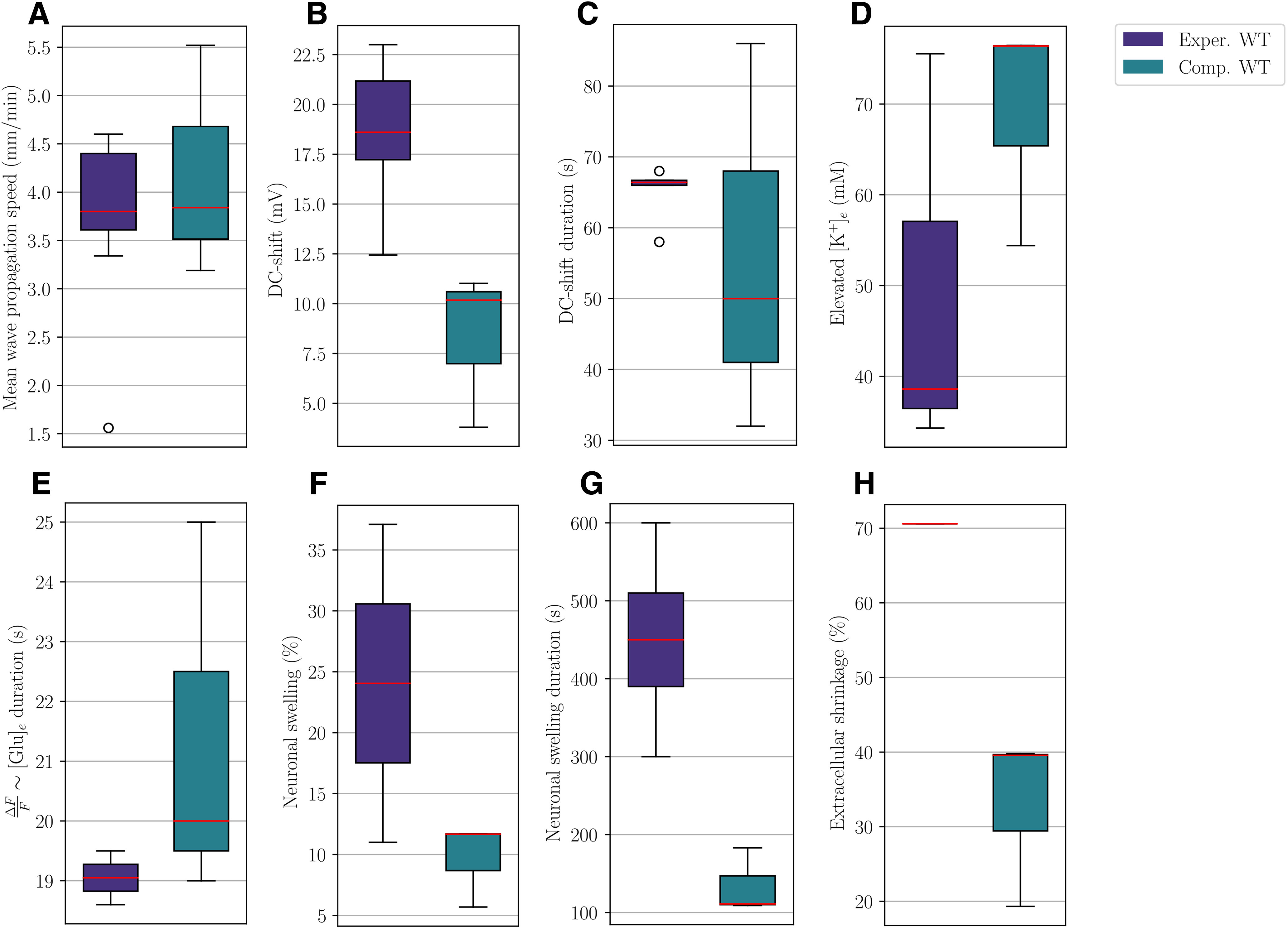
Comparison of intervals (boxes) and min/max-ranges (whiskers) for experimentally and computationally measured values for relevant quantities in WT mice. Red lines indicate median values, and open circles denote outliers. The panels display the mean wave propagation speed (***A***), amplitude (***B***), and duration (***C***) of the DC shift, amplitude of elevated extracellular potassium (***D***), duration of elevated extracellular glutamate concentration (***E***), amplitude (***F***), and duration (***G***) of neuronal swelling, and amplitude of extracellular shrinkage (***H***).

To evaluate the computational predictions, we compare our simulation results from the WT models (A, B, C) with findings from a selection of experimental studies (summarized in Table 3). For the comparison, we define intervals of computational and experimental values for each of the relevant quantities of interest (Fig. 7). The experimental ranges are defined by the 25th percentile and the 75th percentile of the mean values reported in the experimental studies. The computational ranges are defined by the 25th and 75th percentiles of the values from Models A, B, and C. To indicate variability outside the 25th and 75th percentiles, we also define minimum and maximum values (the min/max-range; for details, see Materials and Methods, Validation principles). Values outside the min/max-range are taken to be outliers.

**Table 3:** Summary and overview of experimentally reported propagation speeds, DC shift amplitudes (DC) and their duration (DC dur.), peak in extracellular K^+^ levels (Peak ${\left[{\text{K}}^{+}\right]}_{\text{e}}$), duration of increased relative changes in mean fluorescence ( $\frac{\text{ΔF}}{\text{F}}$ dur.), alteration in neuronal volume fractions (Δαn) and their duration (Δαn dur.), and alteration in the ECS volume fractions (Δαe) during CSD from a selection of studies in either WT mice [Study (WT)] or AQP4 knock-out mice [Study (AQP4– /–)] measured (M) *in vivo* (IV) or in slices (S)

						$\frac{\text{ΔF}}{\text{F}}$ dur.
		Speed	DC	DC dur.	Peak ${\left[{\text{K}}^{+}\right]}_{\text{e}}$		Δ*α*_n_	Δ*α*_n_ dur.	Δ*α*_e_
		(mm/min)	(mV)	(s)	(mm)	(s)	(%)	(min)	(%)
Study (WT)	M								
Lauritzen and Hansen (1992)	IV	3.8 ± 0.9	23 ± 6						
Theis et al. (2003)	S	3.8 ± 0.4	16.8 ± 1.1	58 ± 15	38.6 ± 3				
–	–	4.4 ± 0.3							
Padmawar et al. (2005)	IV	4.4 ± 0.5							
Takano et al. (2007)	IV			66 ± 4			37.1 ± 0.1	8 – 10	
Chang et al. (2010)	IV		18.5	68					
Zhou et al. (2010)	S	1.56 ± 0.24^†^					11.0 ± 0.9^§^	5 – 7	
Thrane et al. (2013)	IV		18.71 ± 2.11		75.54 ± 1.86				
Enger et al. (2015)	IV	3.34 ± 0.1		66.4 ± 3.8		18.6 ± 1.7			
Yao et al. (2015)	IV	3.70 ± 0.1	22 ± 1.8	31 ± 2.4*	34.3 ± 0.8				70.6^**^
–	–			48 ± 4.8*					
Enger et al. (2017)	IV	4.60 ± 0.2		66.7 ± 10.1		19.5 ± 1.3			
Kucharz et al. (2017)	IV		12.44 ± 0.65						
Study (AQP4^– /–^)	M								
Thrane et al. (2013)	IV		12.80 ± 1.16		64.49 ± 1.73				
Yao et al. (2015)	IV	2.90 ± 0.1	19.6 ± 1.4	38 ± 3.6*	35.5 ± 0.7				72.6^**^
–	–			58 ± 2.4*					
Enger et al. (2017)	IV	4.60 ± 0.2		86.0 ± 13.3		15.7 ± 1.2			

* Duration measured at half-maximum amplitude; † speed reported in experiments with TTX, indicated to be 50% of the CSD wave speed without TTX; § deviation from baseline of high ${\left[{\text{K}}^{+}\right]}_{\text{e}}$ perfusion; ** baseline ECS volume fraction differs between WT mice (0.18) and AQP4^– /–^ mice (0.23).

CSD wave propagation speeds in WT mice are reported in the interval [3.61, 4.4] mm/min, whereas the computational WT models give mean wave propagation speeds between 3.52 and 4.68 mm/min (Fig. 7*A*). For the wave speed, the computational and experimental results are thus in good agreement. As for the DC shift, the experimental values are in the min/max-range [12.44, 23] mV, whereas we observe DC shifts within a min/max-range of [3.80, 11.02] mV in the numerical simulations (Fig. 7*B*). We note that the lowest computational DC shift of 3.8 mV (Model B) likely is an underestimate, as we expect some diffusion through the astrocytic networks. We thus find that the computational DC shifts are not in agreement with the experimental values. Regarding the duration of the DC shift, the experimental interval ([66, 66.7] s, excluding measurements at half maximum amplitude) overlap with the computational interval ([41, 68] s; Fig. 7*C*), and the results are thus in good agreement.

The computational values for the peak in extracellular K^+^ concentration overlap with the experimental reports (min/max-range of [54.4, 76.46] vs [34.3, 75.54] mm; Fig. 7*D*). Experimentally, elevated glutamate levels are typically indicated via the relative change in mean fluorescence (ΔF/F) over time (Enger et al., 2015, 2017). Comparing their duration, we find that elevation of extracellular glutamate in our computational models last for 19–25 s (min/max-range), whereas the duration of the increase in ΔF/F has been reported experimentally in a min/max-range of [18.6, 19.5] s (Fig. 7*E*). The ranges of elevated extracellular glutamate duration thus overlap.

Neuronal swelling is reported in the min/max-range [11, 37.1]% in the collection of experimental studies, with a duration of minimum 300 s and maximum 600 s. We observe a neuronal swelling in the min/max-range [5.69, 11.69]%, lasting for 109–183 s (Fig. 7*F*,*G*). The range of computational neuronal swelling amplitudes thus overlap with the experimental range, whereas the computational durations do not agree with experimental reports. We also note that neither Zhou et al. (2010) nor Takano et al. (2007) find that astrocytes swell significantly during CSD. In contrast, our numerical findings show glial (astrocytic) swelling within a range of [4.05, 7.13]%. Regarding extracellular shrinkage, Yao et al. (2015) report a 70.6% reduction of the ECS volume. The computational values are not in agreement: predicting a reduction in the extracellular volume fraction in a min/max-range of [19.32, 39.79]% (Fig. 7*H*).

### Reducing the glial water permeability affects cellular swelling

**Figure 8.**
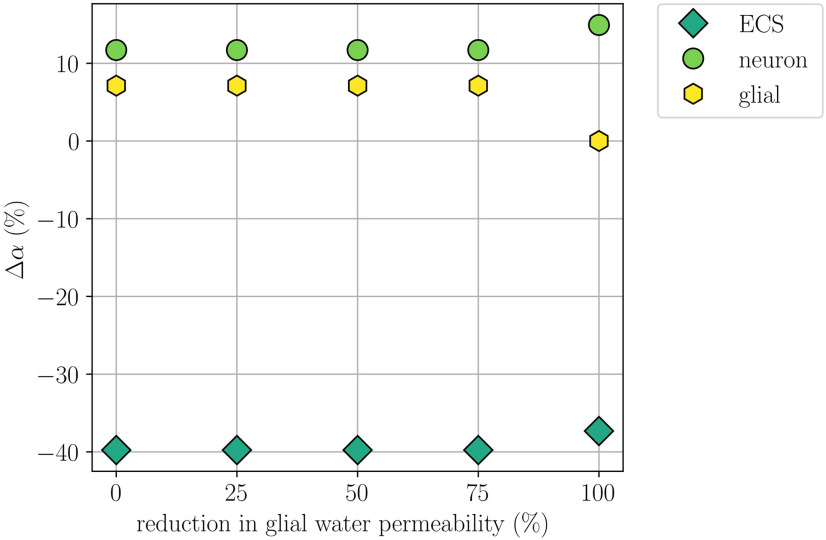
Comparison of changes in volume fractions during simulated CSD where the glial water permeability *η*_ge_ is reduced by 0% (Model A), 25%, 50%, 75%, and 100% (Model D). The panel displays the largest (over time) change in neuronal, glial, and ECS volume fractions relative to baseline at *x *=* *2 mm for each of the reductions.

When setting the glial water permeability to zero, we naturally observe no glial swelling, while the neuronal swelling increases by 27.7% compared with the baseline and the resulting shrinkage of the ECS is 6.2% smaller (Model D; Table 2). The remaining CSD wave characteristics, including the CSD wave propagation speed, do not change notably. Reducing the water permeability by 25%, 50%, or 75% results in unaltered CSD wave characteristics, notably including unaltered cellular swelling (Fig. 8).

Experimentally, Thrane et al. (2013), Yao et al. (2015), and Enger et al. (2017) have studied CSD in AQP4 knock-out mice. Enger et al. (2017) observe no difference in the CSD wave propagation speeds between the WT and AQP4 knock-outs (4.6 ± 0.2 mm/min). This is in contrast to the findings of Yao et al. (2015), who report that the CSD wave propagation speed is reduced by 22% in AQP4 knock-outs. Yao et al. (2015) and Thrane et al. (2013) also report a small, but significant, reduction in the DC shift amplitude in AQP4 knock-outs compared with WT mice, whereas Enger et al. (2017) observe no significant difference in either the duration or the amplitude of the DC shift between AQP4^– /–^ and WT. Enger et al. (2017) additionally study extracellular glutamate elevations during CSD. They report a 20% reduction in the duration of glutamate elevation for AQP4 knock-outs (18.6 ± 1.7 s in WT vs 15.7 ± 1.2 s in AQP4 knock-outs). Moreover, Thrane et al. (2013) report that the amplitude of elevated levels of extracellular K^+^ is significantly lower in AQP4^– /–^ than in WT mice. Our computational findings (modulo the duration of glutamate elevation) are thus in agreement with the experimental results by Enger et al. (2017).

### Reduced K_ir_ 4.1 expression changes glial and DC shift dynamics

**Figure 9.**
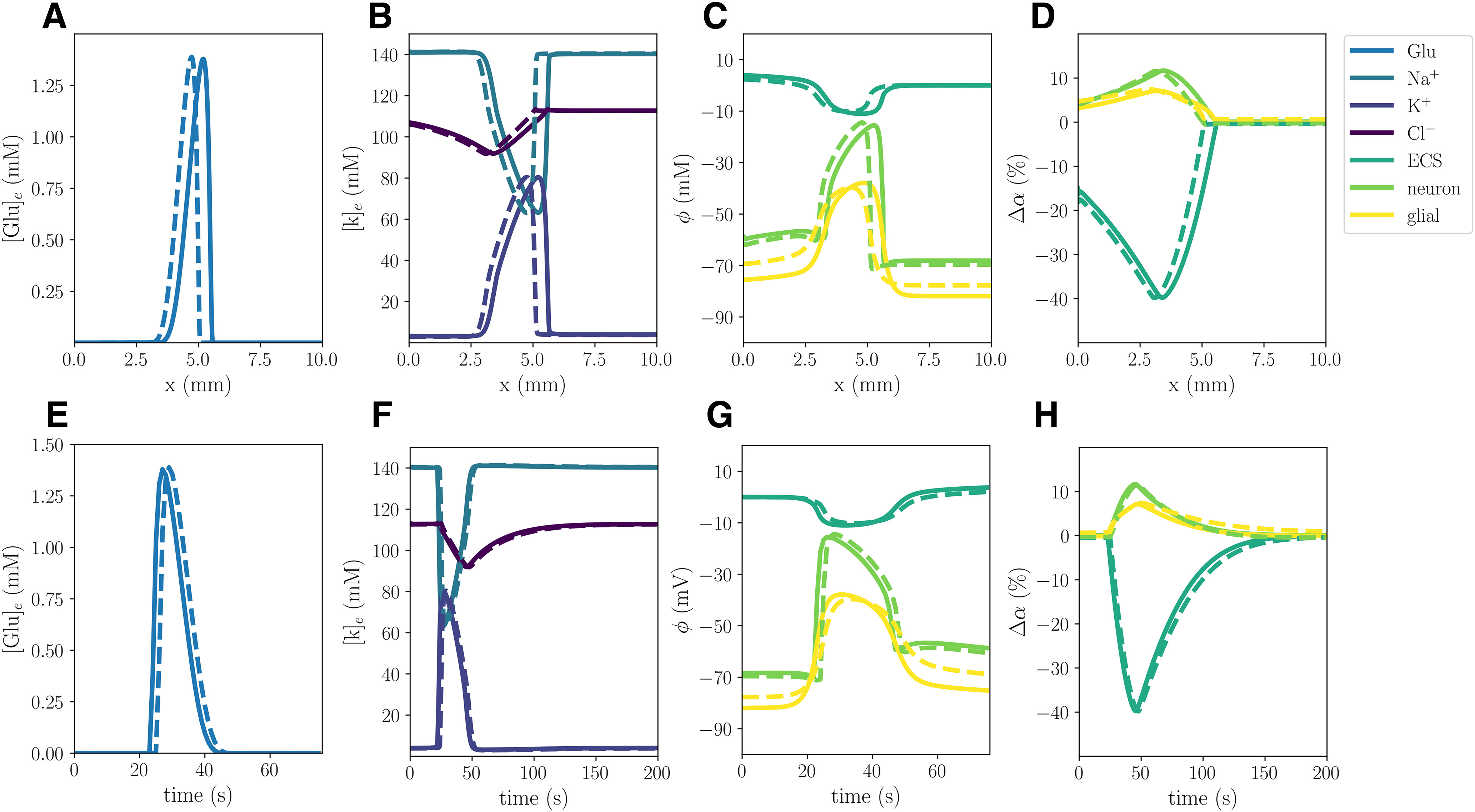
Comparison of Model A (solid) and Model E (stippled) CSD wave. The upper panels display snapshots in time of ECS glutamate (***A***), ECS ion concentrations (***B***), potentials (***C***), and change in volume fractions (***D***) at 60 s. The lower panels display time evolution of ECS glutamate (***E***), ECS ion concentrations (***F***), potentials (***G***), and change in volume fractions (***H***) evaluated at *x *=* *2.0 mm.

**Figure 10.**
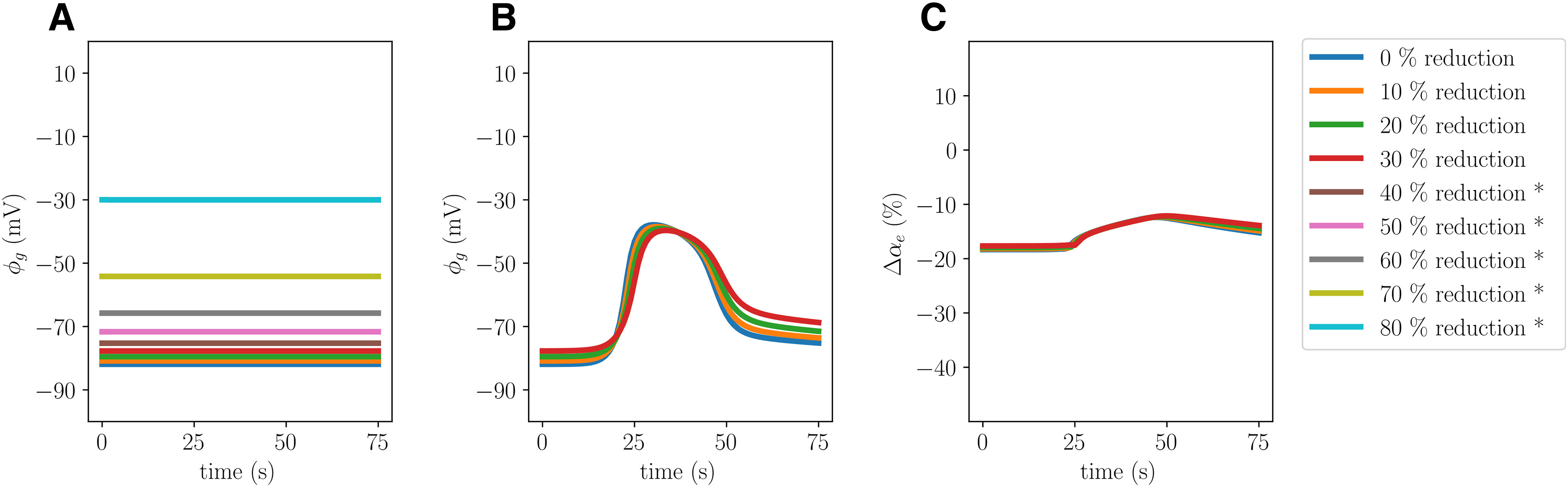
Comparison of glial dynamics without stimuli (***A***) and during induced CSD (***B***, ***C***) where the K_ir_ 4.1 expression is reduced by 0% (Model A), 10%, 20%, and 30% (Model E), 40%, 50%, 60%, 70%, and 80%. The panels display the resting glial potential (***A***), the glial potential during simulated CSD (***B***), and the change in glial volume fractions during simulated CSD (***C***) evaluated at *x *=* *2.0 mm. Asterisks (*) indicate that CSD could not be induced successfully in the computational models.

Reducing the K_ir_ 4.1 channel conductivity by 30% (Model E) alters the CSD wave characteristics, inducing changes in the glial potential, glial swelling and DC shift (Table 2; Fig. 9). In particular, we observe that the glial membrane potential amplitude drops from 55.14 to 48.23 when the K_ir_ 4.1 expression is reduced. Moreover, we observe a 6% reduction in glial swelling amplitude, a 1% increase in neuronal swelling amplitude and a corresponding decrease in the ECS shrinkage amplitude. Reducing K_ir_ 4.1 also results in a slightly higher ECS K^+^ amplitude (0.2%), a slightly lower ECS glutamate amplitude (1%), and a reduced wave speed (9.8%). The computational (nonlinear) solver diverges when CSD is attempted induced in variants for which $g^{{\text{K}}_{\text{ir}}4.1}$ is reduced by 40% or more. However, we observe that the new steady state value for the glial potential increases as we reduce $g^{{\text{K}}_{\text{ir}}4.1}$ (Fig. 10*A*, no CSD induced). An 80% reduction in $g^{{\text{K}}_{\text{ir}}4.1}$ results in a 63% increase in the glia resting potential (from –82 to –30 mV; Fig. 10*A*).

## Discussion

In this study, we have simulated CSD in computational models including multiple triggering mechanisms under variations in morphologic properties, intercellular diffusivity, membrane water permeabilities, and channel conductance, and compared the computational findings with experimental reports. The range of wave speeds, DC shifts duration, peak extracellular K^+^ concentration, neuronal changes in volume fraction, and extracellular shrinkage obtained computationally all overlap with the experimentally reported ranges. Our findings show that intercellular glial diffusion strongly affects the DC shift, and that the ratio of cellular membrane area to tissue volume strongly affects the CSD wave speed. The computational model predicts that a reduced K_ir_ 4.1 expression will reduce the glial swelling and the depolarization of the glial membrane during CSD.

While several papers consider mathematical and computational modeling of CSD (Kager et al., 2000, 2002; Shapiro, 2001; Almeida et al., 2004; Florence et al., 2009; Wei et al., 2014; Mori, 2015; O’Connell and Mori, 2016; Tuttle et al., 2019), few have performed an systematic comparison with experimental literature. The effects of varying glial gap junction strength and the K_ir_ 4.1 conductance on the DC shift and CSD wave speed has previously been computationally assessed by O’Connell and Mori (2016). They find that the glial gap junction coupling impacts the DC shift, which is in agreement with our computational results. Further, they find that the glial cell either buffer or broadcast K^+^, depending on the values of the gap junction and K_ir_ 4.1 conductance. To our knowledge, there are no computational studies examining the effects of reduced glial water permeability or exploring the effect of membrane area-to-volume ratios during CSD.

There are several experimental studies on the role of AQP4 in CSD (Thrane et al., 2013; Yao et al., 2015; Enger et al., 2017; Rosic et al., 2019). Astroglial transmembrane water transport may also occur via other membrane mechanisms (e.g., EAAT, NKCC, VRAC; MacAulay et al., 2004; Pedersen et al., 2016), and the individual contributions to the total transmembrane water permeability of these different mechanisms are debated. Instead of targeting specific water mechanisms, we have here studied the effect of reducing the total glial transmembrane water permeability. When eliminating the glial membrane water transport entirely (Model D), we find, as expected, that the glial cells do not swell at all, whereas the neuronal swelling increases in comparison with the baseline model (Model A). We do not find any difference in CSD wave speed nor in any other computational quantity of interest between these two models. We note that the experimental literature is inconclusive with regard to this point. Our computational findings are largely in agreement with the experimental findings of Enger et al. (2017), knocking out AQP4 does not substantially alter the CSD wave characteristics. In contrast, Yao et al. (2015) and Thrane et al. (2013) report significant differences in their AQP4^– /–^ mice. It is however clear that AQP4 knock-out mice may have additional morphologic differences, e.g., related to the ECS volume: Yao et al. (2008, 2015) report a ∼30% larger ECS baseline volume in AQP4^– /–^ than in WT mice. Our findings suggest that if a reduction in CSD wave speed in AQP4^– /–^ is present, it originates from other factors than the water permeability of the glial membrane. In particular, the ratio between glial and neuronal membrane area and tissue volume was found to be an important factor for the CSD wave speed.

Diffusion of extracellular K^+^ has been hypothesized to be an underlying mechanisms in CSD wave propagation (Grafstein, 1956; Obrenovitch and Zilkha, 1995; Pietrobon and Moskowitz, 2014). Local elevations in ${\left[{\text{K}}^{+}\right]}_{\text{e}}$ will, via diffusion, increase the levels of ${\left[{\text{K}}^{+}\right]}_{\text{e}}$ in neighboring regions, activating voltage-gated and/or ${\left[{\text{K}}^{+}\right]}_{\text{e}}$-dependent channels which leads to a further depolarization of the membrane and thus further release of K^+^ into the ECS. Our models predict extracellular K^+^ amplitudes and wave speeds that are in line with studies performed without the dampening effects of anesthesia; thus, we conjecture that these observations in our computational results may be connected. Indeed, in the model with reduced membrane area-to-volume, we observe lower extracellular K^+^ amplitudes and lower wave speeds.

Spatial K^+^ buffering by astrocytes is hypothesized to be a key mechanism in controlling extracellular K^+^ levels. Astrocytes buffer and redistribute extracellular K^+^ via the astrocytic networks by transferring K^+^ ions from regions with high K^+^ concentration to regions with lower K^+^ levels (Orkand et al., 1966; Kofuji and Newman, 2004). There are open questions related to the role of spatial K^+^ buffering in CSD. Spatial buffering may prevent buildup of extracellular K^+^, or it could potentially increase the CSD wave speed through K^+^ distribution in the direction of the wave propagation. The influx of K^+^ during spatial buffering is mainly mediated by the inward rectifying K^+^ channel K_ir_ 4.1 (Ohno, 2018; Rasmussen et al., 2020). In general, studying the role of K_ir_ 4.1 channels *in vivo* is challenging: the lack of K_ir_ 4.1 has been reported to cause hyperexcitable neurons, epileptic seizures, and premature death (within three to four weeks) in mice (Djukic et al., 2007). Modelling K_ir_ 4.1 knock-outs computationally could potentially aid in investigating the role of K_ir_ 4.1. Our findings show that a reduced K_ir_ 4.1 expression will reduce glial swelling and the depolarization of the glial membrane during CSD. We also observe a slightly reduced CSD wave propagation speed. Further, glial buffering currents have been suggested as an important mechanism behind DC potentials in brain tissue (Herreras and Makarova, 2020; Sætra et al., 2021). This is in line with our observation that a reduction in glial intercellular diffusion reduces the DC shift: reducing glial intercellular diffusion will effectively remove the glial buffering currents.

In terms of limitations, the model framework is founded on an homogenized representation of the tissue: the ECS, the cell membrane and the intracellular space are assumed to exist everywhere in the computational domain. For the spatial and temporal scales involved in the propagation of CSD, such a representation seems appropriate. Next, our simulations are based on a one-dimensional computational domain. As CSD waves spread through the whole depth of the cortical tissue, waves in two or three dimensions would be a more accurate representation. We would expect the computational CSD wave speeds to be reduced in center-initiated two-dimensional simulations. We also note that experimental studies often measure relative fluorescence intensity instead of glutamate concentrations directly, while substantial uncertainty is associated with those that do (Fabricius et al., 1993; Scheller et al., 2000). We have therefore not compared this model quantity with experimental data.

The astroglial membrane expresses a variety of voltage-dependent and leak K^+^ channels (Olsen, 2012), while our mathematical model only accounts for K_ir_ 4.1. The lack of other K^+^ model mechanisms limits the computational range of K_ir_ 4.1 permeabilities: reductions further than 30% leads to numerical instabilities. We hypothesize that a further reduction of the K_ir_ 4.1 expression could be obtained by including other glial K^+^ membrane channels (e.g., K^+^ leak channels). For further computational studies of K_ir_ 4.1 dynamics, it would indeed be advantageous to reduce the K_ir_ 4.1 expression to the point where the resting glial membrane potential is around –58 mV, as observed *in vivo* (Chever et al., 2010). Differences in CSD DC shift duration between different sites have been noted in experiments (Theis et al., 2003; Yao et al., 2015). In our computational model, because of the homogeneity of the material parameters, we expect (and observe) little spatial variation in the computed quantities of interest. Including heterogeneity and/or anisotropy in the model parameters would allow for studying how local tissue properties effect CSD dynamics.

The brain blood supply is continuously adjusted to match the tissue demand. CSD is, however, characterized by a transient supply-demand mismatch, sometimes referred to as vascular uncoupling (Ayata and Lauritzen, 2015). Mestre et al. (2020) demonstrated vasoconstriction associated with anoxic SDs in a stroke model facilitated CSF influx along the perivascular spaces via the so-called glymphatic pathway, and that this fluid was an important source of water accumulation in the ensuing brain edema. Moreover, the hemodynamic responses in CSD varies considerably across species, experimental approach, and more (Ayata and Lauritzen, 2015). An attempt to generalize this response, by for instance mimicking a variable Na^+^/K^+^-ATPase activity or K_ir_ 4.1 conductance because of fluctuating ATP availability, is therefore outside the scope of our computational model.

Validating complex computational models with numerous state variables and model parameters against experimental findings with considerable variability is challenging. Although the methodology suggested here captures variability between the experimental studies, it fails in capturing the uncertainty (i.e., the SD) within each individual study. Conducting rigorous meta-analysis by combining data from the individual experiments, weighted by, e.g., the studies quality, size and/or other factors, to a pooled estimate for the experimental quantities of interest would be advantageous. However, the methodological differences (e.g., recording techniques, recording sites, *in vivo*, awake or anesthetized, vs *in vitro*) between the experimental studies makes meta-analysis difficult (Greco et al., 2013). The computational model considered within this work is complex with numerous model parameters, many of which are difficult to measure experimentally, giving rise to considerable uncertainty. A thorough uncertainty and sensitivity analysis of the computational CSD model would be interesting for future work. Importantly, the model constitutes a general yet detailed framework for capturing fundamental mechanisms of the interplay between ionic movement and volume control of the ECS. As such, applications of the framework are not limited to CSD.

We conclude that the Mori framework is a promising tool for predicting complex phenomena of ionic electrodiffusion and osmotic dynamics in brain tissue. Our findings indicate that the computational model predictions qualitatively matched well with experiments when applied to CSD. As a general framework for ionic dynamics and osmosis, applications are not limited to CSD. Mathematical modeling can be useful for isolating effects and, e.g., pointing at potential confounding effects in AQP4 knock-out mice and how they differ from WT in various aspects.

## Synthesis

Reviewing Editor: Niraj Desai, National Institute of Neurological Disorders and Stroke Decisions are customarily a result of the Reviewing Editor and the peer reviewers coming together and discussing their recommendations until a consensus is reached. When revisions are invited, a fact-based synthesis statement explaining their decision and outlining what is needed to prepare a revision will be listed below. The following reviewer(s) agreed to reveal their identity: Robert Cudmore.

Two reviewers and I have read your manuscript, and we all find it to be well-written, informative, and likely to be useful to the neuroscience community. Our consensus was that manuscript would be strengthened by a more detailed explanation of and justification for the parameter space explored, as both reviewers note in their written reports (which are appended).

In addition, one reviewer offered a careful critique, including several ideas for clarifying the mechanisms of the model and a request . Please go through this critique carefully and respond point-by-point.

REVIEW #1

Introduction

This manuscript presents a computational framework to examine critical parameters in the initiation and resultant features of CSD. It is not clear how this model formulation is different from previous models (Mori, 2015). These differences, if any, need to be addressed.

Model parameters varied include: intracellular diffusion, transmembrane water and K+ permeabilities, and membrane conductances. A major concern is there are only two conditions for each. It would be useful to see at least one of these parameters more systematically varied and model behavior examined. Basically a better examination of model-parameter versus model-behavior space.

The computational model is realistic as it makes meaningful predictions of the majority of experimentally measured phenomena including: wave speeds, DC shift durations, peak in extracellular K+, neuronal changes in volume fraction, and ECS shrinkage. Interestingly, the model is successful in making new predictions that can now be experimentally tested.

Finally, this manuscript provides a novel contribution by a systematic comparison between experimentally derived parameters extracted from a literature review and compares this to the model behavior. This experimental versus model comparison effectively lays the foundation for a framework where experimental and modelling studies can be effectively compared and will endow future models with the ability to make new experimental predictions.

Major Points

Point 1: Is the current model the same as Mori (2015)? If it is, it needs to be stated “it is the same model”. If it is not the same, key differences between the two models need to be enumerated? For example, terms in the equations that were dropped from Mori (2015), terms added, and/or terms modified?

Point 2: All of the manipulations of model parameters are binary. Comparing the wild-type model to one other parameter. Performed by setting values to 0, effectively removing all contributions or by setting a model parameter to a fraction of the wild-type model. For example:

a) Blocking glial gap junctions by setting model parameter to 0 (model B).

b) Examining resultant changes in volume fractions by setting AQP4 to 0 (model D).

c) Reducing K-IR-4.1 by 70% (model E)

Was there any attempt to examine the ‘graded’ response and resulting characteristics of CSD by setting these values to a range like 0, 25%, 50%, 75%, 100% of the wild-type (model A)? If this is done, is there a graded response in model behavior or are there discontinuities? One example would be to determine if there is a graded response in changes in volume fraction during CSD when AQP4 flux is set to a number of intermediate values. I am not asking for a full parameter space exploration as this is prohibitively large. Showing at least one case where the model is robust to varying levels of a parameter would be useful. Basically, show the parameter space is continuous/graded or maybe not, maybe it is defined by abrupt changes?

Point 3: Most of the model perturbations and their effect on CSD features provide positive phenotypes where changes in a model parameter results in changes in CSD properties. It would be good to see the addition of other meaningful biophysical parameters that control ion flux, like Ca++ channels in neurons, and show perturbation of Ca++ flux does not affect the resultant parameters of CSD? This would be a good negative-control modelling experiment.

Point 4: I would like to see a discussion of other model parameters such as fluid delivery and removal by the vasculature. Many CSD studies examine changes in blood-flow in response to CSD. The manuscript needs to address these experimental results in the context of the current model. This needs to be done at least in the discussion.

If tissue is going to swell it needs a source of water, likewise, if tissue is going to shrink it needs a sink for water. The prime candidate of this water source/sink is from the blood plasma which is ultimately mediated by vascular endothelial cell water transport (both active and passive/osmotic).

Is the swelling of cells and the reduction of the ECS itself homeostatic with no overall tissue volume change beyond control? Or, is there an overall increase/decrease in the volume of the sum of neuronal, glial, and extracellular space? In the wild type model, neurons and glia swell by a combined 18% and the ECS shrinks by 40%. This tells me there is an overall 20% reduction in all modelled tissue. Where did that water volume go? An imbalance in the addition or removal process(es) of water by the vasculature is an important parameter that can result in an imbalance and loss of water homeostasis.

For example, in results 3.5 it is noted that ECS shrinkage does not match experimental results. Again, this could be due to a lack of vascular water diffusion in the model? It would be good to address these possibilities in the discussion.

Minor Points

Introduction, “These choices of model scenarios are in part motivated by the incomplete or disparate findings on the role of AQP4 and and Kir4.1 channels.”

Please specify the cell types these channels are in. I am assuming, both AQP4 and Kir4.1 are expressed by Glial cells? Where AQP4 fluxes water and Kir4.1 is an ATP-sensitive K+ channel. Maybe mention that CSD will presumably deplete or at least change ATP levels and have a direct impact on Kir4.1 channel activity. What do we know about this change in ATP levels from the experimental and modelling literature?

Methods 2.1, “The neuronal and glial membrane potentials are defined as the difference between the neuronal and glial, and extracellular potential, respectively.”

Can you simplify this sentence? I am assuming the neuronal membrane potential is defined as the difference between the neuronal and extracellular potentials. Same for the glial membrane potential as the difference between the glial and extracellular potentials. My question is, there is no direct relationship between membrane potentials of neurons and glial? Am I correct?

Methods 2.2, The acronym SD is used but not defined? Is this shorthand for CSD or a different type of generalized spreading-depression different from CSD?

Methods 2.7, Please mention that “FEniCS 2019.1.0” is for solving partial differential equations. It is not part of your computer, processor, or operating system where code was run.

Results 3.3. “Reducing the effective intercellular diffusion (model B)”. Model B removes all intracellular diffusion in astrocytes entirely. Is “reducing” really appropriate here, please consider revising? If the intracellular diffusion were to be varied for 4 different parameter settings and the model behavior examined then using “reducing” would be appropriate.

Results 3.6. “Reducing the glial water permeability affects cellular swelling”. Again, the model is removing glial water permeability entirely, it is potentially misleading to say “reducing” unless this parameter is systematically reduced.

Figure 3. Can the description of the top row “The upper panels display snapshots of ...” indicate they are the spatial response of the model.

Figure 4. Please expand the figure caption to tell the reader what the individual plots are showing (what is the Y-Axis?) and add subplot labels a-h as was done in Figure 3. In particular, the second row, second/third/fourth columns. Also, is “Red lines and dots indicate median values and outliers, respectively” referring to the two open circles as outliers in first row, first and third column? Make those symbols red or refer to them as “open circles”.

Figure 5. Can you run the model with more than two values of the K_IR conductance? More than just the control (wild-type) value and the value you chose for model E (0.390 S/m^2)? Why did you choose that value for model E? Please at least provide a rational for the choice. Are the changes in the model output graded as you vary the K_IR conductance, say by four different values? All the changes you show are small, at least how this is plotted. Would be good to show the model response to say 4 different levels of K_IR conductance to show at least one of these results is graded between the wild-type model to the K_IR value you chose with intermediates in between. As figure 3 showed for models A and C where the changes were large.

Supplemental Methods A.1. Does your model really include “oncotic” pressure? As far as I understand, oncotic pressure is a special type of osmotic pressure and usually refers to proteins in blood plasma that displace water molecules effectively causing a water-deficit which drives water back into the plasma from, say, neural tissue? Please expand how oncotic pressure is included in your equations.

REVIEW #2

In this paper, the authors compare a biophysical model of cortical spreading depression with experimental data. The biophysical model is a coarse-grained model based on electrodiffusion of ions and transmembrane ion/water fluxes. Although the modeling framework was proposed previously and was applied to spreading depression, this work goes further and perform a systematic testing and validation of this framework. The reviewer believes that the paper makes an important contribution, and recommends publication once the following concern is addressed.

The authors use computational results of models A, B and C to compare with the range of experimental measurements. Can the authors provide a rationale for why these choices were made? Given the uncertainties in many of the other parameters, I think it would be better if the authors can provide a stronger justification for using computational runs from the three parameter sets as the theoretical predictions.
